# Microsatellite instability in colorectal adenomas and hyperplastic polyps in Lynch syndrome

**DOI:** 10.1186/1897-4287-9-S1-O4

**Published:** 2011-03-10

**Authors:** Matthew B Yurgelun, Ajay Goel, C Richard Boland, Elena M Stoffel, Investigators of the Great Lakes-New England Early Detection Research Network (GLNE-EDRN)

**Affiliations:** 1 Division of Hematology and Oncology, Department of Medicine, Beth Israel Deaconess Medical Center, Boston, Massachusetts, 02215, USA; 2 Division of Gastroenterology, Department of Internal Medicine, Baylor University Medical Center and Charles A. Sammons Cancer Center, Dallas, Texas, 75246, USA; 3 Division of Gastroenterology, Department of Medicine, Brigham and Women’s Hospital, Boston, Massachusetts, 02115, USA

## Background

Lynch syndrome is characterized by germline mutations in DNA mismatch repair (MMR) genes and carries up to a 70% lifetime risk of colorectal cancer. Impaired MMR gene function results in an abundance of small aberrant nucleotide repeat sequences termed microsatellite instability (MSI). MSI is present in 80-85% of colorectal cancers associated with Lynch syndrome. Prior studies have demonstrated that MMR gene function (as measured by immunohistochemistry) is often lost in Lynch-associated adenomas but is preserved in hyperplastic polyps. The aim of our study was to evaluate the prevalence of MSI in adenomatous and hyperplastic polyps from individuals with Lynch Syndrome.

## Materials and methods

We identified 63 polyps (37 adenomas and 26 hyperplastic polyps) from 34 subjects with known germline MMR gene mutations. Clinicopathological information for each polyp was obtained from retrospective review of pathology reports. MSI analysis was performed on microdissected polyp DNA using pentaplex PCR for a panel of five quasimonomorphic mononucleotide repeat sequences [1]. If ≥2/5 sequences were mutated, the polyp was determined to have MSI.

## Results

MSI was identified in 14/37 (38%) adenomas, compared with 3/26 (12%) hyperplastic polyps (p = 0.021). Prevalence of MSI was significantly higher in larger polyps; among the lesions ≥10mm, 6/7 (86%) adenomas and 1/1 (100%) hyperplastic polyps demonstrated MSI. There was no association between MSI status and patient sex, polyp location, or the patient’s underlying MMR gene mutation. Although MSI appeared more prevalent in adenomas from individuals ≥age 50 years (53% vs 22% for age<50), this did not achieve statistical significance (p = 0.057).

## Conclusions

Overall, MSI was detected in 38% of adenomas and 12% of hyperplastic polyps from individuals with MMR mutations. MSI analysis of small colorectal adenomas would have low sensitivity for identifying patients who should be considered for genetic testing for Lynch syndrome. The finding of MSI in a small fraction of hyperplastic polyps raises questions about their neoplastic potential in patients with Lynch syndrome.
